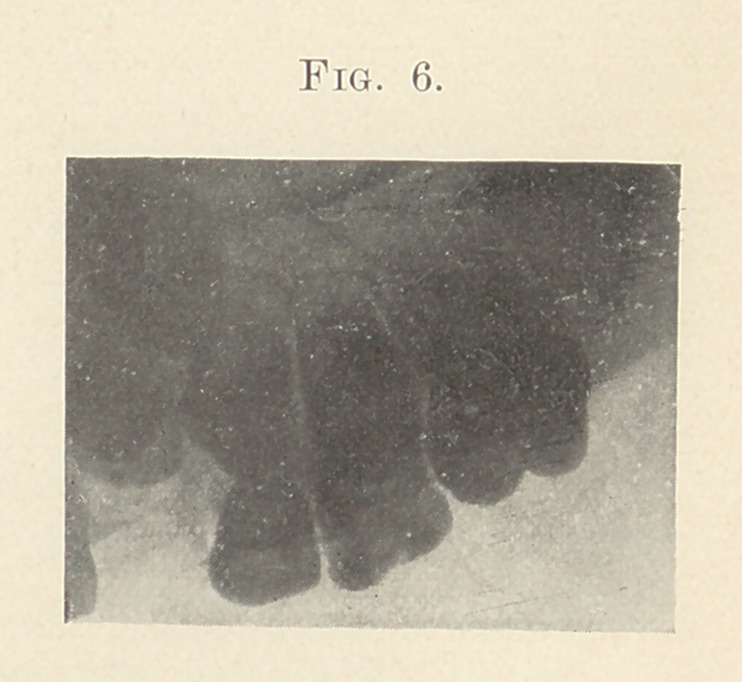# Seventeen Supernumerary Teeth

**Published:** 1900-10

**Authors:** Dwight M. Clapp

**Affiliations:** Boston, Mass.


### International Dental Journal.

Vol. XXI. October, 1900. No. 10.

### Original Communications.[1]

### SEVENTEEN SUPERNUMERARY TEETH.

BY DWIGHT M. CLAPP, D.M.D., BOSTON, MASS.

A very interesting case was recently referred to me by Dr. T. M. Rotch, of Boston, the eminent specialist in children's diseases. The patient, a girl a little under seven years of age, slight, and of a nervous temperament, had never erupted the left temporary cuspid.

Fig. 1, from a photograph of a model of the mouth, shows the absence of this tooth; also the presence of a considerable swelling in the region of the cuspid root.

Figs. 2 and 3 are from X-rays, different positions of the mouth. They show the temporary incisors, their roots being nearly absorbed. In Fig. 3 the permanent cuspid is clearly seen, and both Figs. 2 and 3 show some kind of a tooth corresponding to the permanent lateral; also the permanent bicuspids. Both cuts show the place that should be occupied by the root of the temporary cuspid to be filled with a jumbled-up mass of something corresponding in density to about that of ordinary teeth. What this mass was I could not determine, Fig. 2 showing that it was not connected

[1] The editor and publishers are not responsible for the views of authors of papers published in this department, nor for any claim to novelty, or otherwise, that may be made by them. No papers will be received for this department that have appeared in any other journal published in the country.

with the tooth above, and Fig. 3 that it was probably distinct from the bicuspid.

The half-tone reproductions, although very good, indeed, do not show the same definition that can be clearly seen in the X-ray negatives.

I concluded that an operation was advisable, although there had never been any pain or disturbance of any kind at or near the affected part.

On May 26, 1900, the patient was etherized, and I removed from the swelling the seventeen supernumerary teeth shown in Fig. 4. I have placed beside these teeth an ordinary superior lateral, so that the corresponding size of the supernumerary teeth may be seen. The illustration is almost the exact size of the originals.

The teeth seemed to be each enclosed in a tough, connective-tissue-like membrane, and the whole encysted, something like a bunch of grapes, in a pocket in the bone, there being the merest shell of the alveolus covering the cyst externally. The illustration gives a very correct idea of the shapes of these teeth.

Each is composed of bone and enamel, and has its separate nerve-supply canal.

Figs. 5 and 6 are reproductions of X-rays taken after the removal of the supernumerary teeth.

In Fig. 5 the permanent cuspid is very clearly shown, and the bicuspids are especially well defined in Fig. 6. Both figs., especially Fig. 5, show that the place of the permanent lateral is occupied by what appears to be a perfect bicuspid.

I shall watch with peculiar interest the development of these teeth, and hope in some future number of the JOURNAL to continue their history.

The patient made a rapid recovery, there being no unfavorable symptoms attending the operation.

## Figures and Tables

**Fig. 1. f1:**
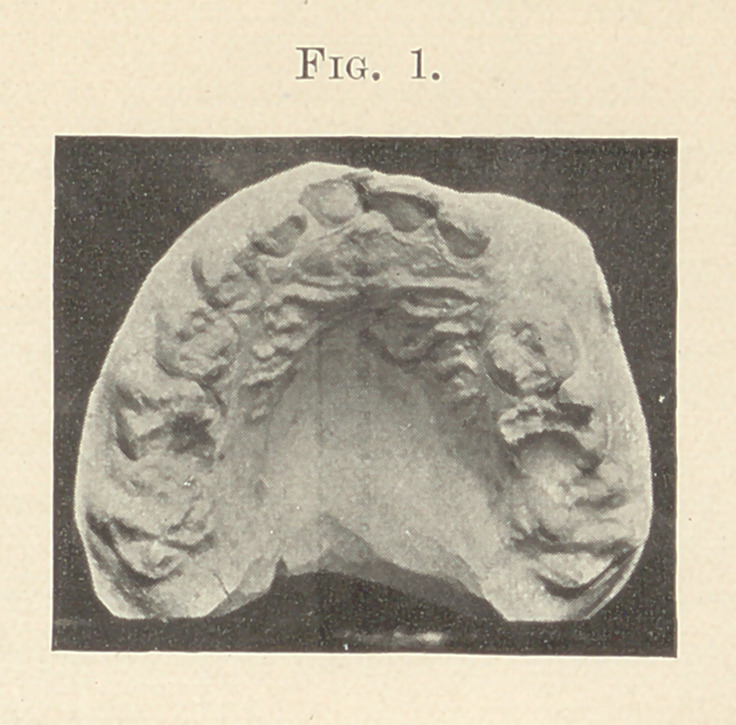


**Fig. 2. f2:**
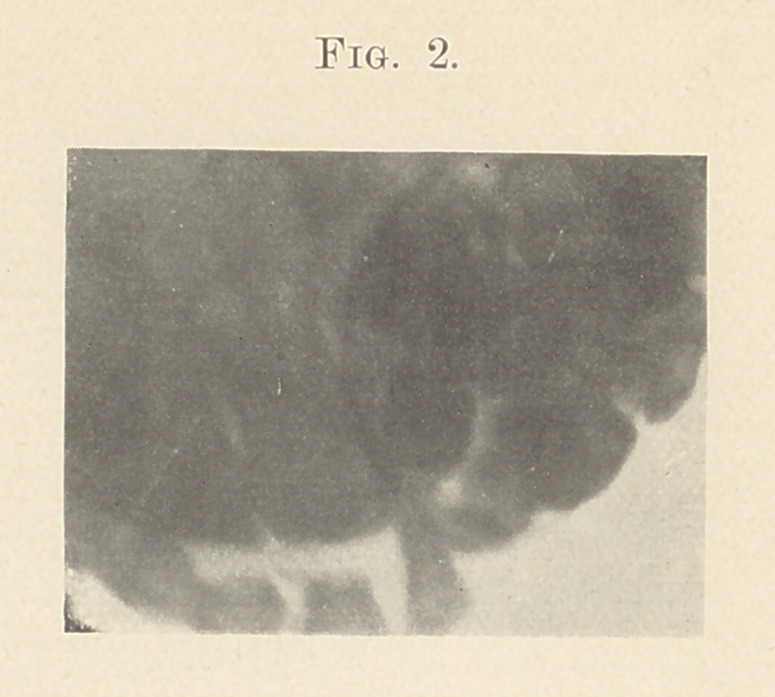


**Fig. 3. f3:**
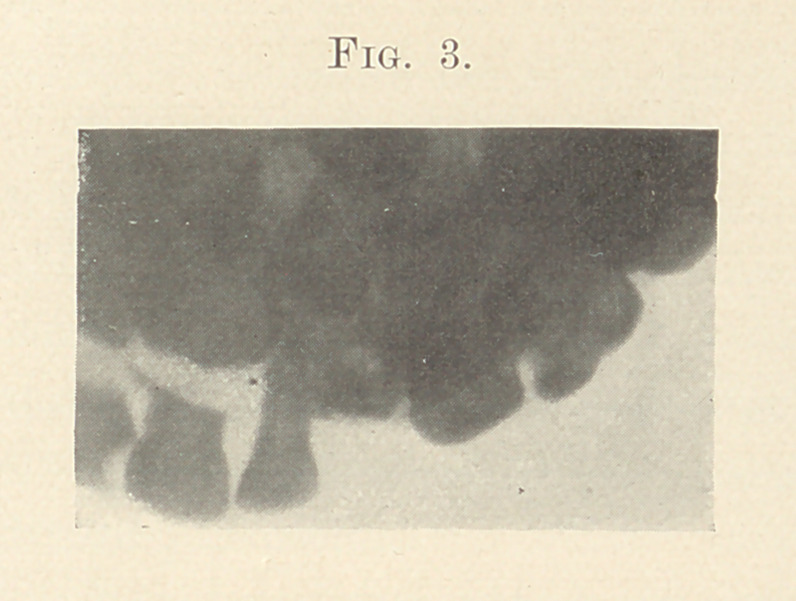


**Fig. 4. f4:**
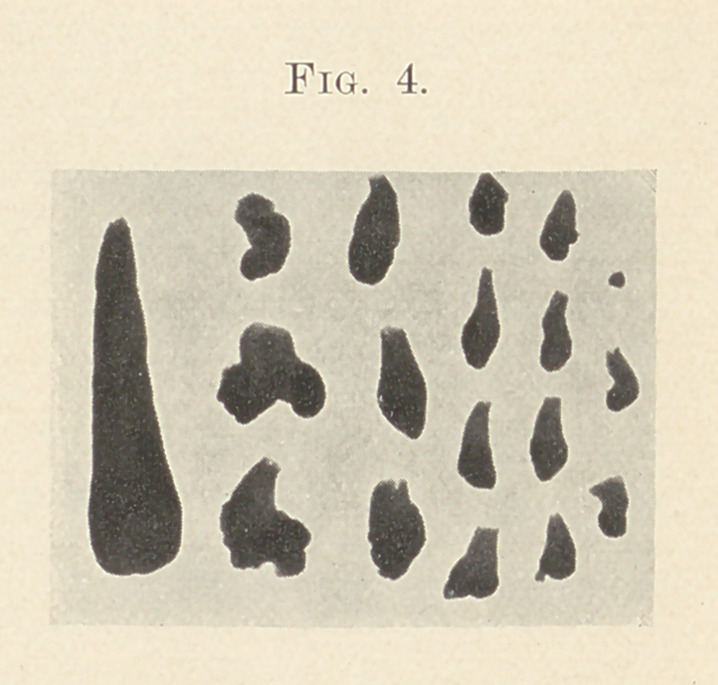


**Fig. 5. f5:**
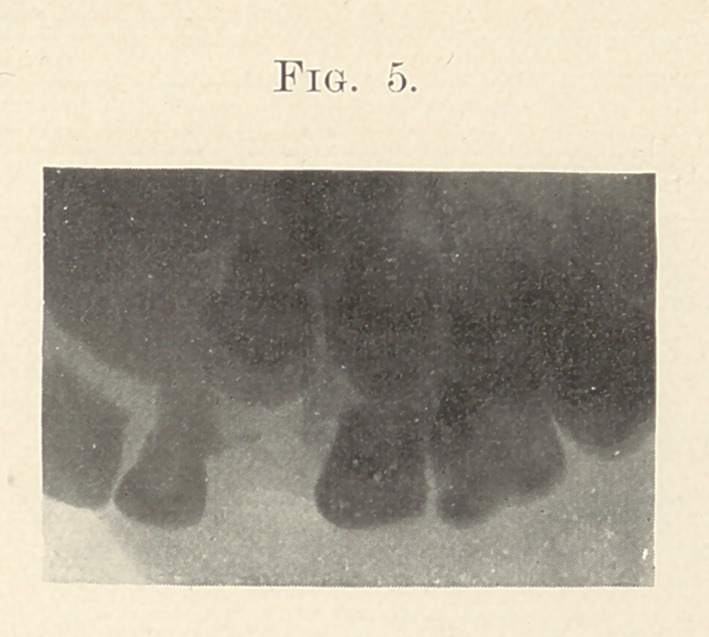


**Fig. 6. f6:**